# Aligning Microtomography Analysis with Traditional Anatomy for a 3D Understanding of the Host-Parasite Interface – *Phoradendron* spp. Case Study

**DOI:** 10.3389/fpls.2016.01340

**Published:** 2016-08-31

**Authors:** Luíza Teixeira-Costa, Gregório C. T. Ceccantini

**Affiliations:** Group of Ecological Wood Anatomy and Parasitic Plant Biology, Department of Botany, Institute of Biosciences, University of São PauloSão Paulo, Brazil

**Keywords:** haustorium, microtomography, endophytic system, Santalaceae, mistletoe

## Abstract

The complex endophytic structure formed by parasitic plant species often represents a challenge in the study of the host-parasite interface. Even with the large amounts of anatomical slides, a three-dimensional comprehension of the structure may still be difficult to obtain. In the present study we applied the High Resolution X-ray Computed Tomography (HRXCT) analysis along with usual plant anatomy techniques in order to compare the infestation pattern of two mistletoe species of the genus *Phoradendron*. Additionally, we tested the use of contrasting solutions in order to improve the detection of the parasite’s endophytic tissue. To our knowledge, this is the first study to show the three-dimensional structure of host-mistletoe interface by using HRXCT technique. Results showed that *Phoradendron perrottetii* growing on the host *Tapirira guianensis* forms small woody galls with a restricted endophytic system. The sinkers were short and eventually grouped creating a continuous interface with the host wood. On the other hand, the long sinkers of *P. bathyoryctum* penetrate deeply into the wood of *Cedrela fissilis* branching in all directions throughout the woody gall area, forming a spread-out infestation pattern. The results indicate that the HRXCT is indeed a powerful approach to understand the endophytic system of parasitic plants. The combination of three-dimensional models of the infestation with anatomical analysis provided a broader understanding of the host-parasite connection. Unique anatomic features are reported for the sinkes of *P. perrottetii*, while the endophytic tissue of *P. bathyoryctum* conformed to general anatomy observed for other species of this genus. These differences are hypothesized to be related to the three-dimensional structure of each endophytic system and the communication stablished with the host.

## Introduction

The complex endophytic structure formed by parasitic plant species renders a rather difficult three dimensional comprehension. Traditional techniques in plant anatomy have been extensively employed to describe the interface formed between parasites and their hosts. However, due to difficulties in imbedding and cutting lignified and large materials, these techniques are usually applied to small samples, which provide a detailed but restricted view of the host-parasite interface. Thus, in order to gain a three-dimensional comprehension of the structure, a huge series of sequential anatomical sections are necessary.

This issue becomes even more relevant when analyzing stem hemiparasites, which often form large woody infestation structures that comprise the interface between these plants and their hosts. In his work on the anatomy of the endophytic system of *Phoradendron flavescens*, Calvin (1967) mentions the analysis of hundreds of anatomical sections in order to follow the endophyte’s extension. In this context, the use of High Resolution X-ray Computed Tomography (HRXCT) arises as a technique that allows general three-dimensional analysis of these complex structures, pushing forward the traditional field of plant anatomy.

This technique consists of using a micro-focused X-ray to illuminate the object while an X-ray detector collects the magnified projection images. As the object rotates inside the equipment, hundreds of images are generated. A powerful computer is than used to stack these images creating a virtual three-dimensional object allowing the analysis of its internal structure^1^. Despite its original development for medical diagnosis (Hounsfield, 1976), computed tomography have long been used for several other scientific purposes (Staedler et al., 2013), including studies in the broad field of Plant Sciences (Heeraman et al., 1997; Kaestner et al., 2006; Brodersen et al., 2012; Gee, 2013; Breteron et al., 2015; Cochard et al., 2015 and others).

Considering these matters, we combined traditional plant anatomy with the HRXCT technique in order to broadly and deeply analyze the endophytic system of *Phoradendron* species. Although several studies have dealt with the haustorial anatomy of this genus (Cannon, 1901; Calvin, 1967; Fineran and Calvin, 2000; Schmid et al., 2011; and others), the large number of species it comprises is reflected in the structural diversity of the endophytic system (Kuijt, 1964, 2003). Based on this proposition, we chose to analyze the endophytic system of two morphologically similar *Phoradendron* species of the Neotropical region.

## Materials and Methods

### Plant Material Sampling

**FIGURE 1:**
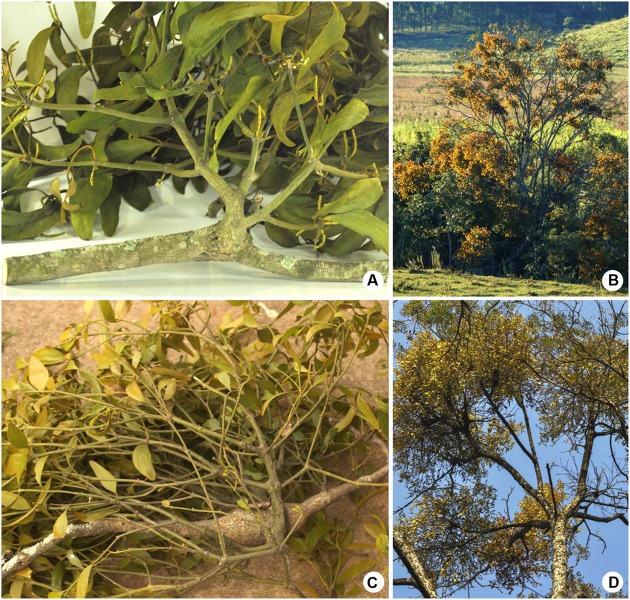
**Two mistletoes of the genus *Phoradendron* and their respective host species. (A,B)** *Phoradendron perrottetii* on *Tapirira guianensis*. **(C,D)** *Phoradendron bathyoryctum* on *Cedrela fissilis*. **(A,C)** Parasitic plants on host branches. **(B,D)** Highly infested trees.

The mistletoe *Phoradendron perrottetii* Nutt. (Santalaceae) growing on branches of the host tree *Tapirira guianensis Aubl.* (Anacardiaceae; **Figure 1A**) was sampled in a riparian forest in Campanha municipality (Minas Gerais state, Brazil). The other mistletoe *P. bathyoryctum* Eichler parasitizing the host tree *Cedrela fissilis* Vell. (Meliaceae; **Figure 1C**) was sampled in a wooded area inside the main campus of the University of Sao Paulo (Sao Paulo state, Brazil). Both parasitic species were capable of colonizing the host canopies, forming moderate to heavy infestations (**Figures 1B,D**).

After sampling we measure the length of all host-mistletoe interface structures – henceforth called “woody galls.” Half of the material was then fixed in a 50% solution of formaldehyde-ethanol-acetic acid (FAA). The other half was air-dried.

**FIGURE 2:**
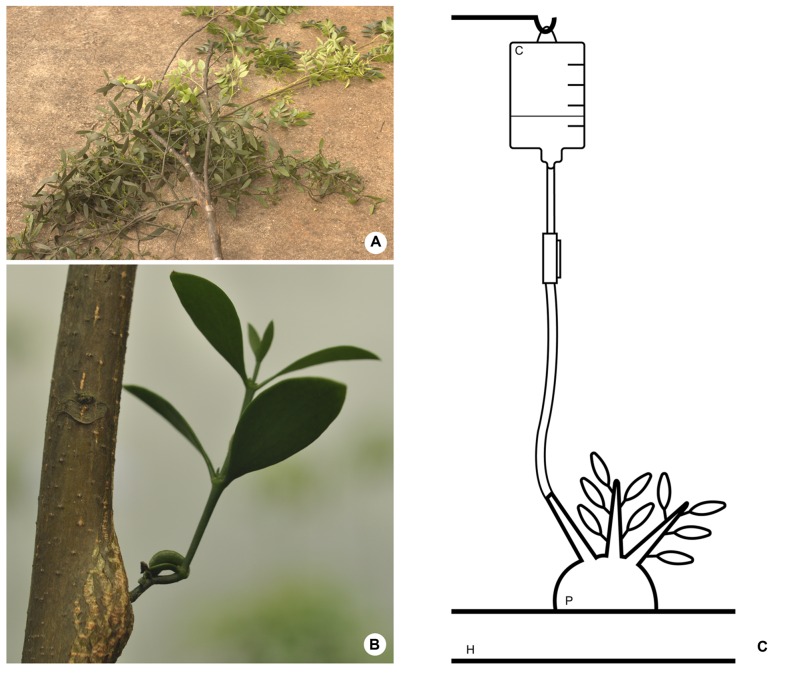
**(A)** *Phoradendron affine* on *Melia azedarach*. **(B)** Mistletoe species (*P. affine*) cultivated on young host tree (*M. azedarach*). **(C)** Schematic drawing of the method employed to perfuse contrasting solutions into the parasitic endophyte. H, host branch; P, parasitic plant; C, contrasting solution.

An additional mistletoe species – *P. affine* (Pohl ex DC.) Engl. and K. Krause (**Figure 2A**) – was cultivated upon young specimens of the host tree *Melia azedarach* L. (Meliaceae; **Figure 2B**). This material was used for the testing of contrasting agents as explained in the next subsection.

Considering the three mistletoe species, we sampled a total amount of 60 woody galls. Several intact woody galls were added to the collection preserved in the Xylarium Nanuza Luiza de Menezes, at Department of Botany of the University of São Paulo (SPFw).

### High Resolution X-ray Computed Tomography Analysis

In order to compare the three-dimensional structure and the infestation patterns of *P. perrottetii* and *P. bathyoryctum* three woody galls formed by each species were scanned using a high performance *in vivo* X-ray microtomography scanner (Skyscan, 1176). Prior to the scanning these woody galls were fixed in a 50% solution of FAA. The fixation aimed on preserving the structure of the endophyte, which are usually composed by abundant parenchyma cells (Thoday, 1957; Calvin, 1967; Kuijt, 2003).

Previous trials were also carried out in order to evaluate other preservation methods, such as air-drying and embedding of the samples. Indeed, the first method was not efficient in preserving the structure of the endophyte. On the other hand, the embedding medium (polyethyleneglycol) was observed to severely interfere with the X-ray penetration into the samples.

Additionally, two woody galls formed by *P. affine* were used to test whether the use of contrasting agents could improve the visualization of the parasite’s endophytic system within the host branches. Based on the work of Staedler et al. (2013) two contrasting solutions were tested – Lugol’s solution (0.1%) and a lead nitrate (PbNO_3_) solution (0.2%). A third sample was not perfused with contrasting agents, thus serving a test control.

Contrasting solutions were applied to the woody galls according to the method described by Sperry et al. (1988) (**Figure 2C**) for vascular infusions. Briefly, one end of a rubber tube was fitted at one of the branches of the parasite close to woody gall. The other end of the tube was connected to an elevated reservoir (0.5 – 1 m) containing the contrasting solution. This set up was designed so that the flexibletube would be filled with the contrasting solution forming a liquid column with enough pressure to force the entry of the solution into the host branch. Special care was taken to avoid the presence of air bubbles in the tube that could be pushed into the xylem and clog the system. Fine surfacing of the wood with a razor blade was also needed to assure that vessels were open and permeable. After being perfused with contrasting solutions for ca. 8 h the material was disconnected from the apparatus and scanned immediately.

The scanning of each woody gall generated hundreds of X-ray images which were subsequently reconstructed using the NRecon software in order to provide a three-dimensional visualization. Analysis and image acquisition were carried out by using the software Dataviewer (two-dimensional analysis of internal structures) and the software Dataviewer (three-dimensional analysis).

### Morphological and Anatomical Analyses

The same woody galls used for HRXCT analyses were also used for morphological and anatomical analyses as a way to provide a detailed understanding of host-mistletoe interface. One material of each host-mistletoe pair was air-dried, cut in transversal and longitudinal sections, and sanded using sand papers of ascending grifts until a smooth surface was obtained. The material was studied and photographed using a stereo-photomicroscope (Leica DML and camera DFC 310FX).

The two remaining woody galls of each mistletoe were used for anatomical analysis. Samples were imbedded in polyethyleneglycol (PEG) and sectioned in a sliding microtome (Leica SM 2000R) to produce 20 μm thick transverse, radial longitudinal, and tangential longitudinal sections, which were stained with safranin/Astra-blue (Johansen, 1940; Bukatsch, 1972 adapted by Kraus and Arduin, 1997).

Additional material of host-mistletoe pair was used to complement the analysis. A total amount of 10 woody galls was studied through stereo-photomicroscope, while five woody galls were studied through plant anatomy techniques.

## Results

### Optimizing the Parameters for HRXCT at the Host-Mistletoe Interface

The use of HRXCT technique for analyzing the three-dimensional host-mistletoe interface began by testing a total of nine scanning parameters. Among these, the voltage and the current had to be adjusted for each sample. **Table 1** lists the adjustment used for each sample along with sample size, duration of each scan, and information about the resulting files.

**Table 1:** Scanning parameters adjustment for each sample scanned using the High Resolution X-ray Computed Tomography technique.

Mistletoe-host pair	Species ID	Sample size (cm)	Voltage (kV)	Current (μA)	Scan duration	Number of files	File size (GB)
*Phoradendron perrottetii* on *Tapirira guianensis*	1	4.70	45	397	00:41:40	515	58
	2	5.80	55	630	01:04:31	721	37
	3	5.43	35	456	01:08:55	730	52
*Phoradendron bathyoryctum* on *Cedrela fissilis*	1	10.56	50	500	03:19:31	1201	402
	2	7.30	62	403	02:27:24	983	108
	3	7.02	46	540	02:31:25	990	103
	
*Phoradendron affine* on *Melia azedarach*	Control						
	
	1	6.00	45	397	01:38:38	815	39
	
	Lugol’s solution						
	
	2	5.90	37	396	01:26:21	802	39
	
	PbNO_3_ solution						
	
	3	5.75	35	375	01:26:16	711	33

After the initial test other relevant parameters, such as resolution, filter, and rotation step were fixed for all samples. Thus, all samples were scanned with maximum resolution (9 μm), using a 0.2 mm aluminum filter and a 0.3° rotation step. Despite the increase on scan duration, maximum resolution, and low rotation steps were used to allow a detailed observation of the parasite’s endophytic system. Due to longer scan duration all samples were previously wrapped in thin plastic film to avoid wood desiccation.

### Three-Dimensional Structure of the Endophytic System

During the sampling and the measurement of the woody galls *P. bathyoryctum* was observed to form larger infestation structures on branches of *C. fissilis* when compared to *P. perrottetii* on branches of *T. guianensis*. These observations served as an initial clue to understanding the extension of the endophytic system of each mistletoe species. The HRXCT technique allowed us to assess not only the endophyte’s extension but also its spread pattern within the host branch.

**FIGURE 3:**
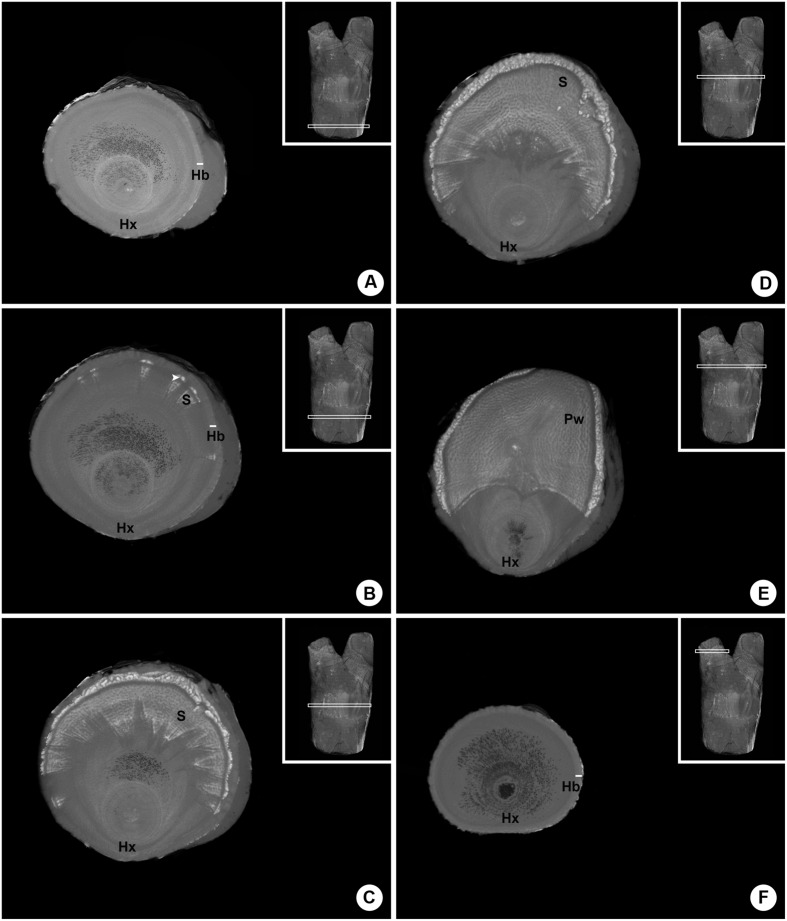
**Sequence of six internal images of the woody gall formed by *P. perrottetii* on the host *T. guianensis*. (A)** Proximal end of the host branch free of parasitic tissue. **(B)** Wedge-shape sinkers penetrating the host wood. **(C)** Increased amount of sinkers and cortical strands. **(D)** Grouping of sinkers. **(E)** Parasitic tissue occupying the upper half of the branch circumference. **(F)** Distal end of the host branch free of parasitic tissue. Hx, host xylem; Hb, host bark; S, sinker; Pw, parasitic wood; white arrow-head, cortical strands.

A series of six internal images of the woody gall formed by *P. perrottetii* illustrates the interface between this mistletoe and the host *T. guianensis* (**Figure 3**). The images taken from proximal and distal ends of sample showed the host branch with no sign of infestation (**Figures 3A,F**, respectively). In the actual region comprising the endophyte (**Figures 3B–E**) parasitic tissue was observed to spread both within the wood and the bark of the host. Initially, few wedge-shape sinkers were observed (**Figure 3B**). As the number of sinkers increased, the amount of cortical strands also increased (**Figure 3C**). The sinkers progressively clustered into the parasite’s wood (**Figure 3D**), which then occupied half of the branch circumference (**Figure 3E**).

**FIGURE 4:**
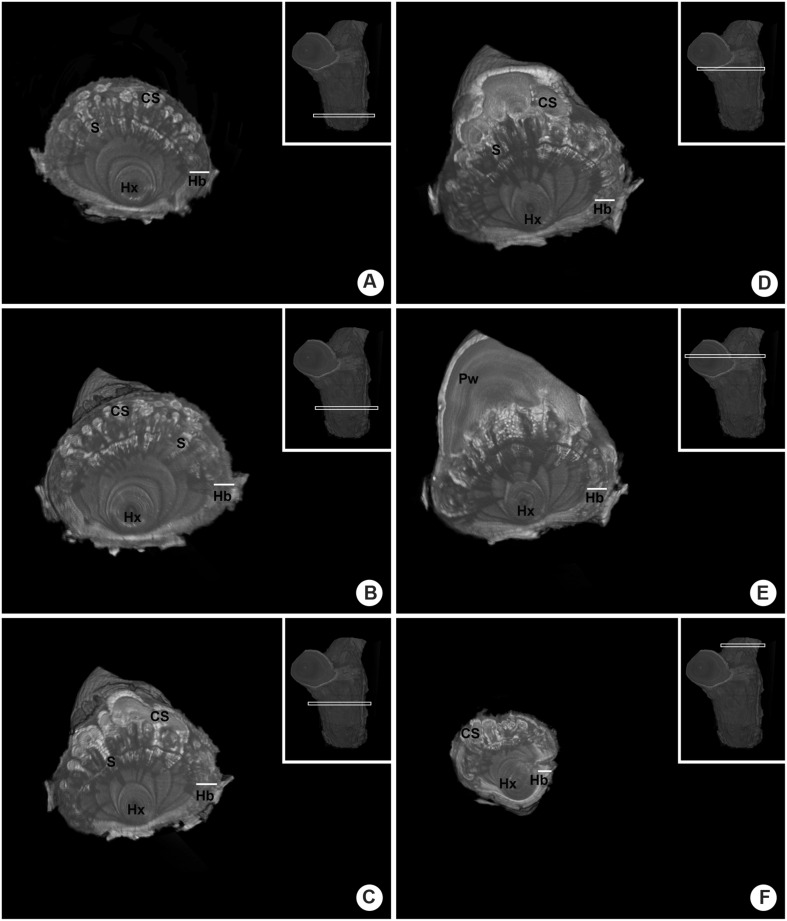
**Sequence of six internal images of the woody gall formed by *P. bathyoryctum* on the host *C. fissilis*. (A)** Proximal end of the host branch already showing infestation by parasitic tissue. **(B)** Large cortical strands associated with elongated sinkers. **(C)** Initial clustering of cortical strands. **(D)** Further clustering of cortical strands. **(E)** Parasitic tissue associated to the parasite’s wood. **(F)** Distal end of the host branch still showing infestation by parasitic tissue. Hx, host xylem; Hb, host bark; CS, cortical strands; S, sinker; Pw, parasitic wood.

A similar series of images was obtained for the woody gall of *P. bathyoryctum* on *C. fissilis* (**Figure 4**). In this case, the parasite’s endophyte extended within the host branch from one end to the other (**Figures 4A–F**). Large cortical strands were associated with elongated sinkers (**Figure 4A**). The cortical strands grew larger (**Figure 4B**) and began to cluster (**Figures 4C,D**) until the parasite’s wood becomes visible (**Figure 4E**). Unlike the endophyte of *P. perrottetti*, only the cortical strands of *P. bathyoryctum* are eventually grouped into its wood as the sinkers remain separated.

The analysis of the three-dimensional internal structure of both parasites’ endophytic systems revealed two opposite patterns. The sinkers of *P. perrottetii* did not penetrate deeply into the host wood and the endophytic tissue was usually restricted to half of the branch circumference forming a concise endophytic pattern. As for *P. bathyoryctum*, its longer sinkers projected throughout the woody gall area in all directions forming a spread out pattern. Please refer to Supplementary Material for short videos that provide a three-dimensional internal view of the structure formed by each parasite.

### Anatomy of the Host-Parasite Interface

**FIGURE 5:**
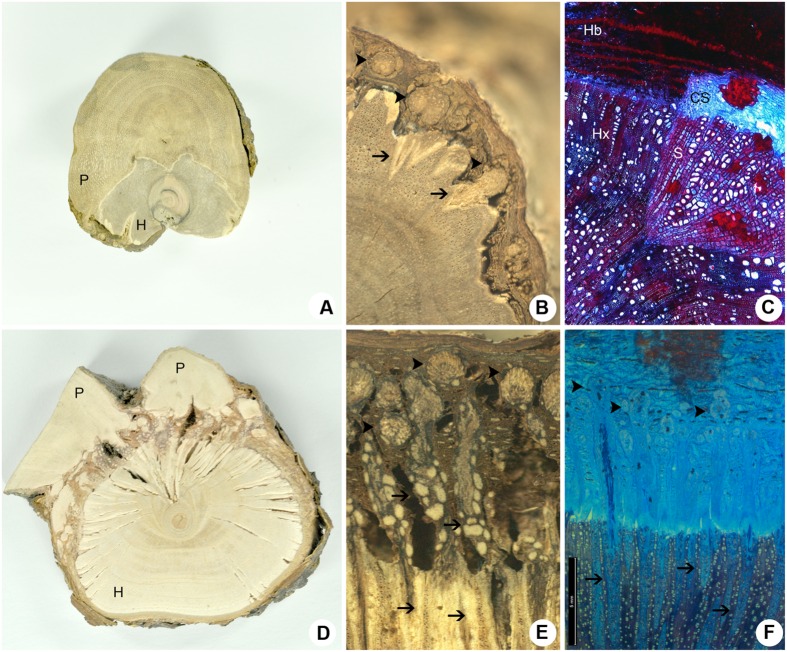
**Comparative cross-sections of the host-parasite interfaces. (A–C)** *P. perrottetii* on *T. guianensis*. **(D–F)** *P. bathyoryctum* on *C. fissilis*. **(A,D)** Macroscopic cross-sections showing the maximum size observed for the parasite’s primary haustorium. **(B,E)** Detail of the macroscopical cross-section showing sinkers arising from cortical strands. **(C,F)** Microscopy cross-section showing the general anatomy of cortical strands and sinkers. Arrow-heads, cortical strands; Arrows, sinkers; CS, cortical strand; H, host; Hb, host bark; Hx, host xylem; P, parasite; S, sinker.

Despite their morphological similarities, *P. perrottetii* and *P. bathyoryctum* showed remarkable differences regarding their endophytic structure and anatomy (**Figure 5**). A transversal section of the woody gall shows the haustorium of the parasitic species *P. perrottetii* forming a continuous interface with the host branch of *T. guianensis* (**Figure 5A**). In this old infestation, the parasite encircled the host branch almost completely forming sinkers around the whole circumference of the host branch. The endophyte was composed of small cortical strands of the parasite, which were observed to give rise to short wedge-shaped sinkers that penetrate into the host xylem (**Figures 5B,C**).

On the other hand, the transverse section of the woody gall formed by *P. bathyoryctum* on branches of *C. fissilis* showed a fragmented interface due to the diffuse organization of the endophytic system (**Figure 5D**). The contact between the mistletoe and the host tissue is accomplished by the multiple cortical strands that spread out through the thick host bark of *C. fissilis* (**Figure 5E**) and give rise to a large number of long needle-shaped sinkers (**Figure 5F**).

**FIGURE 6:**
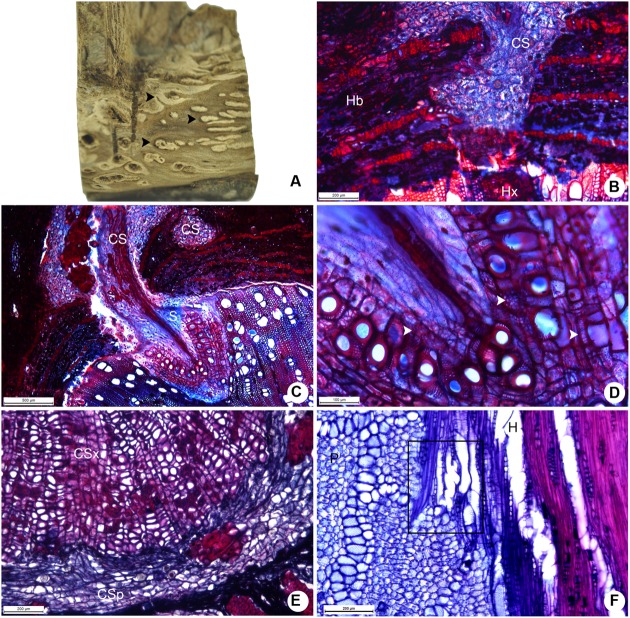
**Aspects of the host-parasite interface between *P. perrottetii* and *T. guianensis*. (A)** Macroscopic view of the woody gall after removal of host bark, showing cortical strands on host xylem. **(B)** Young cortical strand penetrating the host xylem (scale bar = 200 μm). **(C)** Developed cortical strand giving rise to a sinker (scale bar = 500 μm). **(D)** Detail of the sinker showing lignified parenchyma cells (scale bar = 100 μm). **(E)** Cortical strand showing secondary growth (scale bar = 200 μm). **(F)** Direct vessel connection between parasite and host (scale bar = 200 μm). Black arrow-heads, cortical strands; white arrow-heads, lignified parenchyma cells; CS, cortical strand; CSp, phloem tissue of the cortical strand; CSx, xylem tissue of the cortical strand; H, host; Hb, host bark; Hx, host xylem; P, parasite; S, sinker.

Despite being smaller than the cortical strands of *P. bathyoryctum*, the strands formed by *P. perrottetii* on the host tree *T. guianensis* caused morphological alterations to surface of host xylem (**Figure 6A**). At the beginning of its penetration into the host tissues, the cortical strand is composed almost exclusively of parenchyma cells (**Figure 6B**). Later on during its growth, tick-walled fibers and vessels elements are developed in the strands (**Figure 6C**). The vessel elements in the sinker are connected to vessel coming from the cortical strand (**Figure 6D**). A reduced number of cortical strands were observed to undergo secondary growth in *P. perrottetii* (**Figure 6E**). Direct vessel contact between the parasite and the host was also observed (**Figure 6F**).

**FIGURE 7:**
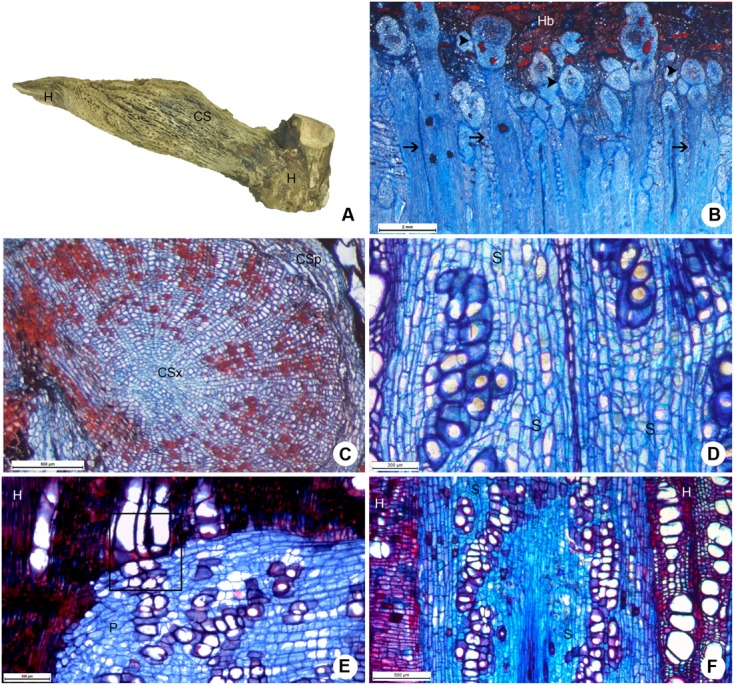
**Host-parasite interface between *P. bathyoryctum* and *C. fissilis*. (A)** Macroscopic view of the woody gall after removal of host bark, showing cortical strands on host xylem. **(B)** Multiple cortical strands in different developmental stages within the host bark (scale bar = 2 mm). **(C)** Cortical strand showing secondary growth (scale bar = 500 μm). **(D)** Detail of the sinker showing lignified parenchyma cells and vessel elements (scale bar = 200 μm). **(E)** Direct vessel connection between parasite and host (scale bar = 200 μm). **(F)** Region of the host xylem with cross-grained wood close to the sinker (scale bar = 500 μm). Arrows, sinkers; arrow heads, cortical strands; CS, cortical strand; CSp, phloem tissue of the cortical strand; CSx, xylem tissue of the cortical strand; H, host; P, parasite; S, sinker.

The large cortical formed by *P. bathyoryctum* on the host tree *C. fissilis* were observed to cause severe alterations to the host wood (**Figure 7A**). The large number of cortical strands allowed us to observe these structures in different growth stages (**Figure 7B**), from primary to secondary growth (**Figure 7C**).

The needle-shaped sinkers were composed of lignified parenchyma which encircled short vessel elements (**Figure 7D**). Few lignified vascular parenchyma cells were also present. Direct vessel contact between the parasite and the host was also observed (**Figure 7E**). The formation of cross-grained wood was observed in some areas of the host xylem close to the sinkers (**Figure 7F**).

Additional images showing the morphology and anatomy of the host-mistletoe interface were compared with images obtained via HRXCT analysis, as suggested by Steppe et al. (2004). These figures are provided as Supplementary Material.

### The Use of Contrasting Agents in HRXCT

**FIGURE 8:**
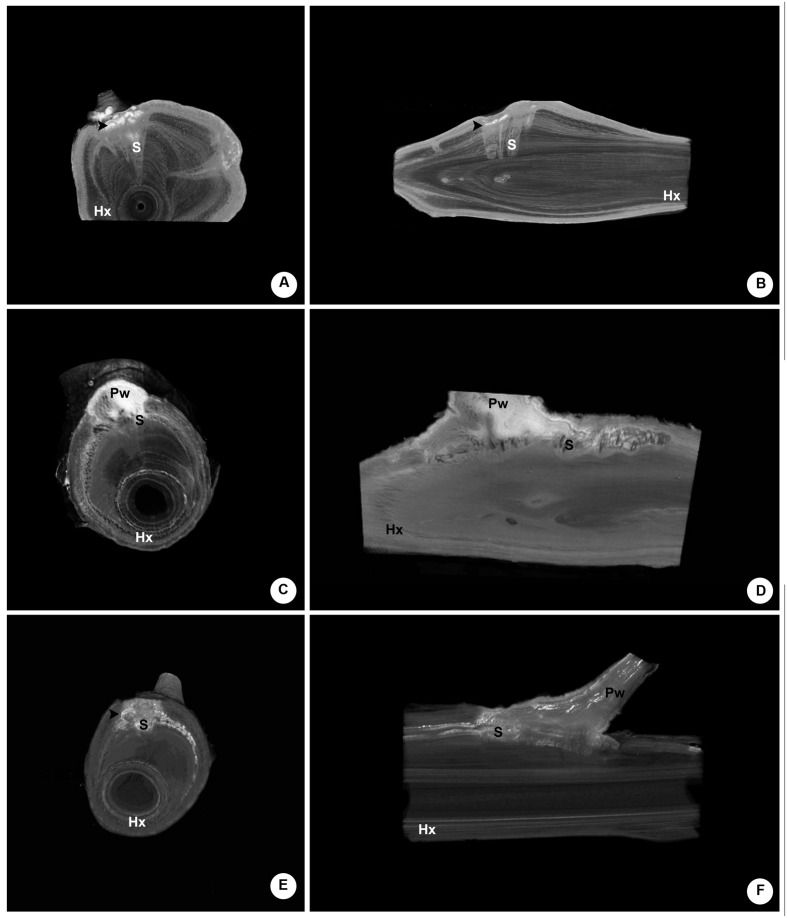
**Comparative internal images of the woody gall formed by *P. affine* on the host *M. azedarach* showing the test of contrasting solutions. (A,B)** Control sample (no contrasting solution) showing cortical the natural contrast of the cortical strands as opposed to the sinkers. **(C,D)** Sample perfused with Lugol’s solution showing the parasitic tissue with a higher contrast. The whole extension of the cortical strand is visible, along with short parasitic sinkers, host vessels and a wood knot. **(E,F)** Sample perfused with lead nitrate solution showing the parasitic tissue with a higher contrast, especially within the vessels of the parasite and the host. Hx, host xylem; S, sinker; Pw, parasitic wood; black arrow-head, cortical strands.

The endophytic tissue of *P. perrottetii* and *P. bathyoryctum* showed a natural contrast against the wood of *T. guianensis* and *C. fissilis*, respectively. In both cases the entire parasitic endophyte was observed in white, while the host wood showed dark shades of gray. However, this was not entirely the case for *P. affine* parasitizing *M. azedarach*. The cortical strands of *P. affine* actually showed natural contrast against the host wood, but the sinkers was seen as light gray structures penetrating the wood of *M. azedarach* (**Figures 8A,B**).

The use of contrasting agents was observed to improve the contrast between the endophytic tissue of the parasite and the wood of the host. In the sample perfused with Lugol’s solution both the cortical strands and the sinker showed a higher contrast (**Figure 8C**). When lower density tissue was removed from the image, the whole extension of the cortical strand became visible in bright white along with short parasitic sinkers, host vessels and a wood knot (**Figure 8D**).

The use of lead nitrate solution also provided a higher contrast to the parasitic tissues and to some of the host’s vessels as well (**Figure 8E**). Nevertheless, the contrasting solution was observed only within the vessels as the surrounding parasitic tissue was again observed in shades of gray (**Figure 8F**).

It’s noteworthy that the control sample had longer and more developed sinkers when compared to the other two samples perfused with contrasting agents. Although all mistletoe seeds were applied to host branches on the same day, their morphological and anatomical development rates varied greatly. Still, it was possible to compare the structure endophytic tissue among the samples.

## Discussion

As other member of the “Viscaceae” clade (currently within the Santalaceae family; Stevens, 2001), *Phoradendron* species attach to their hosts via a single connection (primary haustorium) that frequently form swollen regions referred by Many (1964) as woody galls. Their endophytic system is conspicuous and extensive, which is considered to be a feature of evolutionary derived species due to an increased protection conferred to the parasitic tissue (Heide-Jorgensen, 2008). The endophytic tissue is generally organized in cortical strands that grow longitudinally within the host bark and give rise to sinkers, which then penetrate the host xylem (Calvin, 1967).

However, minor variations to this general organization are observed in some species, such as *P. perrottetii*. In this species an eventual fusion of sinkers occurs and the distinction between sinkers and cortical strands becomes unclear, forming a continuous endophytic tissue and connects to the host. This peculiarity was also observed by Thoday (1957), who analyzed *P. perrottetii* growing on the host *Protium insigne* (Burseraceae). The uniqueness of this endophytic system could be related to preferred, although not restricted, use of a host species. At the study site *P. perrottetti* was mostly observed to use *T. guianensis* as its host despite the presence of other tree species. This host preference was also recorded^2^ for other areas of the Cerrado (Brazilian savanna).

On the other hand, the endophytic anatomy of *P. bathyoryctum* showed more generalized features commonly reported for other *Phoradendron* species (Kuijt, 2003). A great number of mature cortical strands are also observed. Regarding host selection, *P. bathyoryctum* has been reported to grow on species from a total of 14 different plant families showing little predilection for hosts within the Sapindaceae and Meliaceae family^2^.

Not only had the anatomy of the endophytic tissue differed between the studied species, the three-dimensional internal pattern of each parasite’s endophytic system was also diverse. To our knowledge, this is the first study to show the three-dimensional structure of host-mistletoe interface by using HRXCT technique.

While *P. perrottetii* formed small woody galls with a concise endophytic restricted to one area of the host branch, *P. bathyoryctum* had a spread out pattern of the endophyte with longer sinkers that grew throughout the area of the woody galls. The understanding of these internal patterns was readily achieved through the X-ray tomography analysis.

Given its non-destructive method, we chose to begin our study by employing the tomography technique. The high-resolution images and three-dimensional models obtained for the woody galls allowed us to select the most interesting area of the host-parasite interface to be used in the following anatomical analysis eliminating the need for a long series of anatomical sections.

Higher resolutions are also possible, as showed by Dhondt et al. (2010) who made images of *Arabidopsis thaliana* at the cellular level. Nevertheless, due to scan duration and size of generated files, this increased resolution would require a previous reduction of the host-mistletoe interface. This reduction would result in loss of the whole three-dimensional pattern.

Additionally to guiding subsequent anatomical studies, the understanding of the endophytic spread allowed us to better understand the host-parasite communication. Although lumen-to-lumen contact was observed for both host-mistletoe pairs, the sinkers of *P. perrottetii* had a greater amount of vessel elements when compared to the sinkers of *P. bathyoryctum*. The observation may be related to spread of each endophytic system. The spread-out sinkers of *P. bathyoryctum* could compensate the reduced amount of vessel elements by increasing the contact area with the host xylem. Future *in vivo* analysis, such as those carried out by Brodersen et al. (2010) and Cochard et al. (2015) should help addressing this hypothesis.

The host-parasite communication was also indirectly assessed by the use of contrasting solutions. When analyzing the infestation pattern of each parasitic species the high contrast of the endophytic tissue against the host wood was an important factor during the analysis. This was due to the different density between the endophyte and the host tissue. Therefore, the low natural contrast between the sinkers of *P. affine* and the wood of *M. azedarach* was probably related to a similar density of these structures. As observed by other authors (Metscher, 2013; Staedler et al., 2013), the use of contrasting solutions proved to be an efficient way to improve the analysis of this situation.

Each contrasting solutions employed here had an interesting result. Lugol’s solution perfused to the whole endophytic tissue of *P. affine* due to the presence of iodine, which bound to starch grains previously observed within the parasite’s endophyte (data not shown). Host vessels close to the endophyte were also perfused with this solution highlighting the vascular communication between host and parasite. The same occurred in the specimen perfused with lead nitrate solution. However, this solution was only observed within vessels, thus indicating an apoplastic connection between the two plants.

In synthesis, the use of High Resolution X-ray Computed Tomography analysis is proven to be an important non-invasive tool for the understanding of the connection and organization between hosts and their parasites. However, up to the current technology and computing development the use of traditional methods of plant anatomy are still needed for a comprehensive understanding of these complex structures. We are currently testing the use of the HRXCT, as well as the use of contrasting solutions in other host-parasite models.

## Author Contributions

This work is part of the research conducted by LT-C during her Master’s Degree course. Therefore, LT-C was responsible for: experimental planning; all the preparations mentioned in this manuscript; result analysis and discussion; manuscript writing. GC was the Advisor during the conduction of the research presented in this manuscript. Therefore, GC was responsible for: funding obtainment, experimental planning, discussion of results and manuscript writing.

## Conflict of Interest Statement

The authors declare that the research was conducted in the absence of any commercial or financial relationships that could be construed as a potential conflict of interest.

## Footnotes

1 Information on the HRXCT technique available at: http://www.bruker-microct.com/company/methods.htm

2 According to herbaria data available at http://inct.splink.org.br/ (INCT – Virtual Herbaria of Flora and Fungi).

## Supplementary material

VIDEO S1 **Internal view of the host-parasite interface between *P. perrottetii* and *T. guianensis* using the HRXCT technique**.

VIDEO S1 **Internal view of the host-parasite interface between *P. bathyoryctum* and *C. fissilis* using the HRXCT technique**.
