# Supplementary material for: Aligning Microtomography Analysis with Traditional Anatomy for a 3D Understanding of the Host-Parasite Interface – Phoradendron spp. Case Study
Source: Front Plant Sci. 2016 Aug 31;7:1340. doi: 10.3389/fpls.2016.01340 (PMC5006639; doi:10.3389/fpls.2016.01340)
Supplement: Supplementary file 3 [file Image_1.PDF]

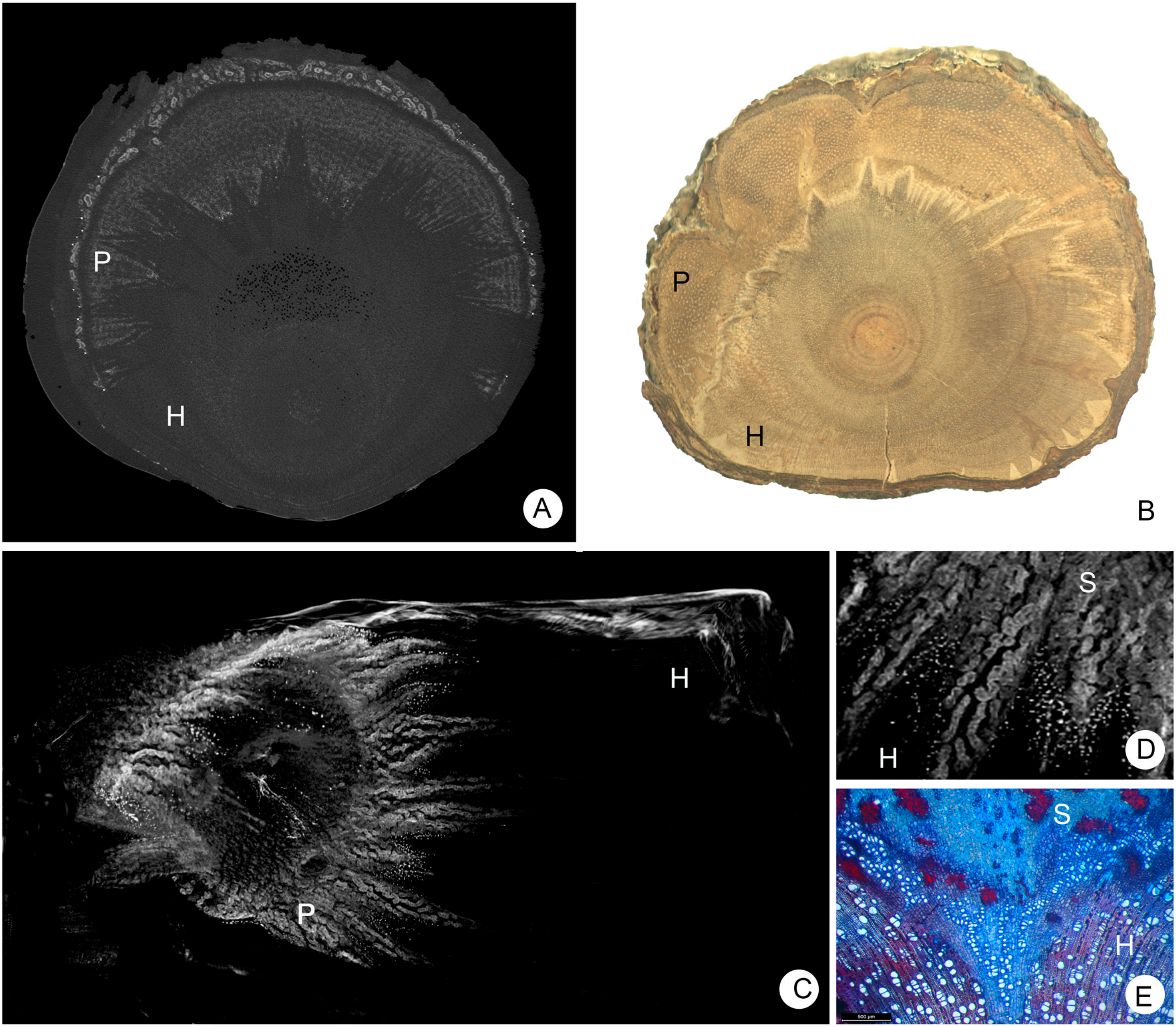

Supplementary Figure 1: Internal images of the woody gall formed by *Phoradendron perrottetii* on*Tapirira guianensis* compared to morphological and anatomical sections. **(A)** Cross-section of the host-parasite interface showing the parasitic endophyte in white and the host wood in grey. **(B)** Macroscopical cross-section of the host-parasite interface showing the parasitic tissue and the host wood. **(C)** Longitudinal section of the host-parasite interface showing the three-dimensional position of the sinkers (host tissue removed from the image). **(D)** Detail of the sinker showing vessels of the parasite. **(E)** Anatomical cross-section of the sinker showing vessels of the parasite (scale bar = 500 µ). H = host; P = parasite; S = sinker.
