# Supplementary material for: Aligning Microtomography Analysis with Traditional Anatomy for a 3D Understanding of the Host-Parasite Interface – Phoradendron spp. Case Study
Source: Front Plant Sci. 2016 Aug 31;7:1340. doi: 10.3389/fpls.2016.01340 (PMC5006639; doi:10.3389/fpls.2016.01340)
Supplement: Supplementary file 4 [file Image_2.PDF]

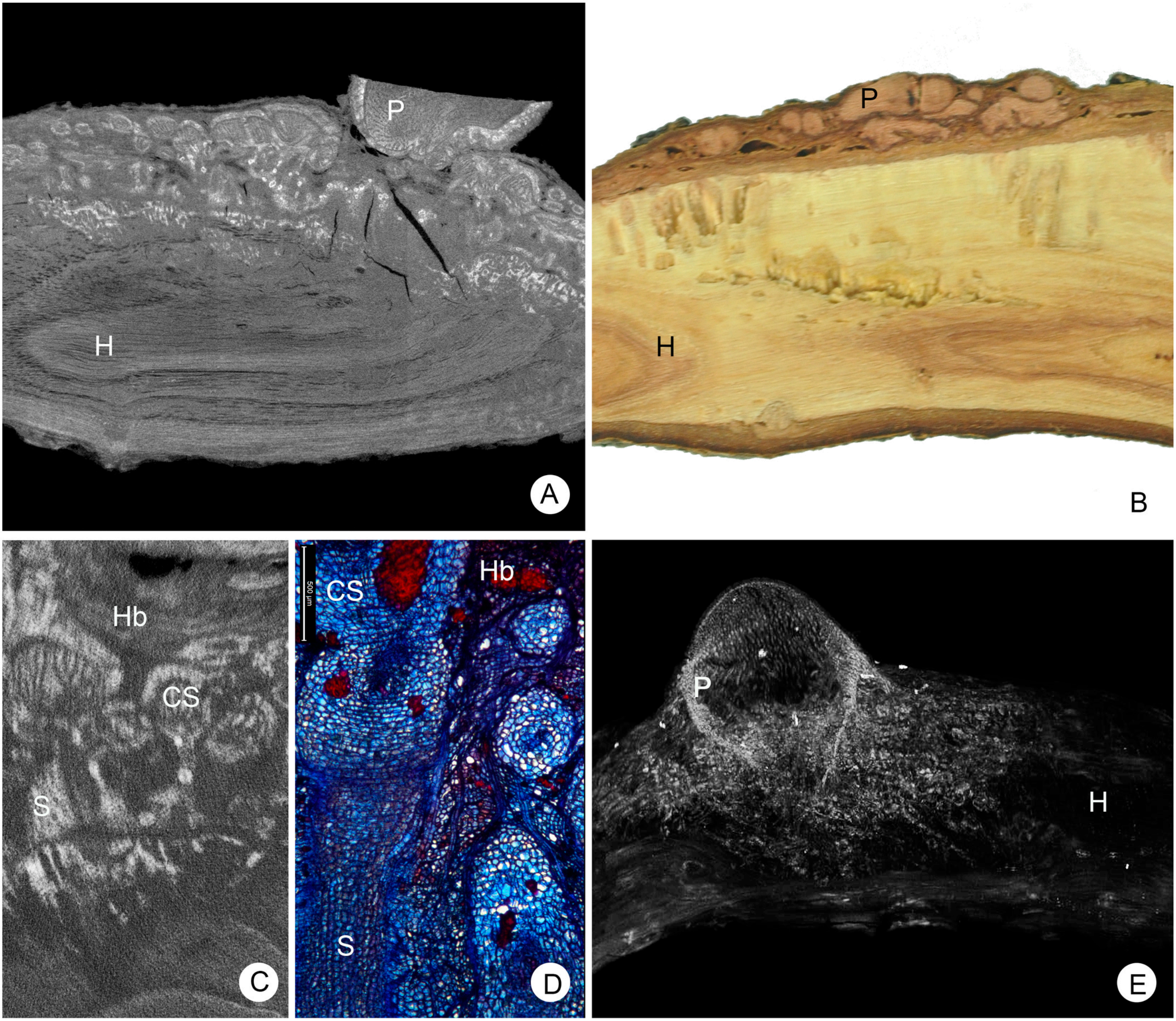

Supplementary Figure 2: Internal images of the woody gall formed by *Phoradendron* bathyoryctum *on Cedrela fissilis.* **(A)** Longitudinal section of the host-parasite interface showing the parasitic endophyte in white and the host wood in grey. **(B)** Macroscopical longitudinal section of the host-parasite interface showing the parasitic endophyte and the host wood. **(C)** Detail of the cortical strands and sinkers within the host bark. **(D)** Anatomical cross-section of the cortical strands and sinkers within the host bark (scale bar = 500 µ). **(E)** Longitudinal section of the host-parasite interface showing the three-dimensional spread of the parasitic endophyte through the host branch. H = host; P = parasite; Hb = host bark; CS = cortical strand; S = sinker.
